# Italian survey about intraperitoneal drain use in distal pancreatectomy

**DOI:** 10.1007/s13304-024-01987-0

**Published:** 2024-10-13

**Authors:** Nicolò Pecorelli, Claudio Ricci, Alessandro Esposito, Giovanni Capretti, Stefano Partelli, Giovanni Butturini, Ugo Boggi, Alessandro Cucchetti, Alessandro Zerbi, Roberto Salvia, Massimo Falconi, Pan-Drain study group, Alberici Laura, Aleotti Francesca, Alfieri Sergio, Angrisani Marco, Anselmo Alessandro, Bannone Elisa, Barabino Matteo, Belfiori Giulio, Belli Andrea, Belli Giulio, Bonatti Chiara, Borgia Gianluca, Caccamo Lucio, Campra Donata, Caputo Damiano, Casadei Riccardo, Cescon Matteo, Citterio Davide, Colangelo Ettore, Colledan Michele, Coppola Roberto, Crippa Stefano, Dall’Olio Tommaso, De Carlis Luciano, De Giorgi Donato, De Luca Raffaele, Del Vecchio Antonella, Della Valle Raffaele, Di Benedetto Fabrizio, Di Dato Armando Di Domenico Stefano, Giovanna Di Meo, Di Sebastiano Pierluigi, Ettorre Giuseppe Maria, Fogliati Alessandro, Frena Antonio, Gavazzi Francesco, Giacomo Batignani, Gianotti Luca, Giuliante Felice, Grazi Gianluca, Grottola Tommaso, Gruttadauria Salvatore, Ingaldi Carlo, Isabella Frigerio, Izzo Francesco, La Barba Giuliano, Langella Serena, Lionetto Gabriella, Lombardi Raffaele, Maganuco Lorenzo, Maggino Laura, Malleo Giuseppe, Manzini Lorenzo, Marchegiani Giovanni, Marchetti Alessio, Marcucci Stefano, Massani Marco, Mastrangelo Laura, Mazzaferro Vincenzo, Mazzola Michele, Memeo Riccardo, Milanetto Anna Caterina, Mocchegiani Federico, Moraldi Luca, Moro Francesco, Napoli Niccolò, Nappo Gennnaro, Nardo Bruno, Pacilio Carlo Alberto, Paiella Salvatore, Papis Davide, Patriti Alberto, Patrono Damiano, Prosperi Enrico, Puglisi Silvana, Ramera Marco, Ravaioli Matteo, Rocca Aldo, Ruzzente Andrea, Sacco Luca, Scialantrone Grazisa, Serenari Matteo, Tamburrino Domenico, Tatani Bruna, Troisi Roberto, Veneroni Luigi, Vivarelli Marco, Zanello Matteo, Zanus Giacomo, Zingaretti Caterina Costanza, Zironda Andrea

**Affiliations:** 1 Pancreatic Surgery Unit, Pancreas Translational and Clinical Research Center, San Raffaele Scientific Institute, Milan, Italy; 2 Vita-Salute” San Raffaele University, Milan, Italy; 3 Department of Internal Medicine and Surgery (DIMEC), Alma Mater Studiorum, University of Bologna, Bologna, Italy; 4 General and Pancreatic Surgery Department, The Pancreas Institute-University of Verona Hospital Trust, Verona, Italy; 5 Pancreatic Surgery Unit, Humanitas Clinical and Research Center-IRCCS, Via Manzoni 56, Rozzano, Milan Italy; 6 Department of Biomedical Sciences, Humanitas University, Via Rita Levi Montalcini 4, Pieve Emanuele, Milan, Italy; 7 Surgical Department, HPB Unit Pederzoli Hospital, Peschiera Del Garda, Italy; 8 Division of General and Transplant Surgery, University of Pisa, Pisa, Italy; 9 Morgagni E Pierantoni Hospital, Forlì, Italy

**Keywords:** Drainage, Distal pancreatectomy, Survey, Questionnaire, Regret, Nicolò Pecorelli

## Abstract

Intraperitoneal prophylactic drain (IPD) use in distal pancreatectomy (DP) is still controversial. A survey was carried out through the Italian community of pancreatic surgeons using institutional emails, Twitter, and Facebook accounts of the Italian Association for the Study of the Pancreas (AISP) and the Italian Association of Hepato-biliary-pancreatic Surgery (AICEP). The survey was structured to learn surgeons’ practice in using IPD through questions and one clinical vignette. Respondents were asked to report their regrets for omission and commission regarding the IPD use for the clinical scenario, eliciting a scale between 0 (no regret) and 100 (maximum regret). The threshold model and a multilevel mixed regression were built to identify respondents’ attitudes. One hundred six surgeons completed the survey. Sixty-three (59.4%) respondents confirmed using at least 1 drain, while 43 (40.6%) placed 2 IPDs. Only 13 respondents (12.3%) declared a change in IPD strategy in patients at high risk of clinically relevant postoperative pancreatic fistula (CR-POPF), while 9 (9.4%) respondents changed their strategy in low-risk POPF situations. Thirty-five (35.5%) respondents declared they would remove the IPD within the third postoperative day (POD) in the absence of CR-POPF suspicion. The median omission regret, which proved to be the wrong decision, was 80 (50–100, IQR). The median regret due to the commission of IPD, which turned out to be useless, was 2.5 (1–20, IQR). The CR-POPF probability threshold at which drainage omission was the less regrettable choice was 7% (1–35, IQR). The threshold to perceive drain omission as the least regrettable choice was higher in female surgeons (*P* < 0.001), in surgeons who modulated the strategies based on the risk of CR-POPF, and in high volume centers (*p* = 0.039). The threshold was lower in surgeons who performed minimally invasive distal pancreatectomy (*P* < 0.001), adopted a closed system (*P* < 0.001), placed two IPDs (*P* < 0.001), or perceived the IPD as important to prevent reintervention (*p* = 0.047). Drain management after DP remains very heterogeneous among surgeons. The regret model suggested that IPD omission could be performed in low-risk patients (7% of CR-POPF), leading to low regret in the case of the wrong decision, making it an acceptable clinical decision.

**Supplementary Information:**

The online version contains supplementary material available at 10.1007/s13304-024-01987-0.

## Introduction

Morbidity rate after distal pancreatectomy (DP) is mainly due to the occurrence of postoperative pancreatic fistula (POPF), which accounts for about 50% of all complications after DP [1]. Intraperitoneal prophylactic drain (IPD) has been considered by pancreatic surgeons as one of the most important strategies to mitigate the clinically relevant POPF (CR-POPF) [2]. The advocated advantages of IPD could be early recognition of POPF-related complications [3], such as post-pancreatectomy hemorrhage (PPH) [4] or the treatment of peripancreatic abscesses by removing infected pancreatic juice from the peritoneal cavity. A couple of recent RCTs [5, 6] demonstrated that IPD omission is safe and may lead to reduced postoperative CR-POPF, especially in the setting of a low-risk pancreatic stump. Nonetheless, despite the high level of evidence, the drain-less approach remains an under-practiced strategy in real life [7]. Several factors could contribute to the uncertainty, such as the lack of a standardized and worldwide accepted tool to predict the risk of CR-POPF (e.g., as Callery’s [8] Fistula risk score) or a standardized approach to managing pancreatic stump after DP [9].

This uncertainty produced a very heterogeneous use and management of IPD after DP in high-level centers [7]. The aim of the present study was to capture the customs of pancreatic surgeons in the Italian community. Moreover, a regret-based decision model was used to elicit the impact of emotional intelligence in decision-making.

## Materials and methods

### Survey

An online survey was sent in June 2022 to the Italian community of pancreatic surgeons using the online platform Survey Planet®. Surgeons affiliated with the Italian Association for the Study of the Pancreas (AISP) and the Italian Association of Hepato-biliary-pancreatic Surgery (AICEP) were asked using the email, the Twitter and Facebook accounts of AISP and the WhatsApp channel of AICEP. All the participants were asked to send separately an e-mail as confirmation of participation if they wished to verify the presence of duplicated answers. All questionnaire responses were mandatory, and each question could not be changed once selected to avoid bias. The engagement rate was established by comparing the number of answers with the number of surgeon members affiliated with AISP and AICEP at the closure of the survey.

The Surgical Task Force of AISP set up 22 questions about the use of drainage in DP: 14 with multiple choice answers, 3 with a visual analogic scale, and 3 open-ended questions (Supplementary file).

The questions included general information about the participants, such as gender, age, and professional level (resident, fellow, or expert surgeon). We also collected information about the type of hospital collected: country, the institutional volume of pancreatic resection, and, if present, other types of institutional surgical activities (colorectal resection, liver resection, upper gastro-intestinal, or others). About the use of IPDs, we asked: i) the number and type of drains for DP); ii) the timing and indications for drain removal, and iv) the motivations behind the choices using a visual analogic scale (0–10). The study was structured following the COREQ standards for reporting qualitative research [10]. Ethical approval was not sought for the present study because of its survey nature.

### Regret model

A vignette was displayed to participants to measure their regret when choosing drain placement. The clinical case was represented by a 70-year-old patient with pancreatic body-tail adenocarcinoma in excellent general conditions who underwent standard laparoscopic DP; the pancreatic thickness was <10 mm at the line transection. Based on their knowledge and experience, pancreatic surgeons were asked to report their regret consequent to the loss of opportunity in the CR-POPF mitigation if the drain was not placed, as well as the regret consequent to the placement of a useless drain. Thus, the *regret of omission* here was the regret felt by the surgeon who omitted the IPD in a patient who otherwise may have benefited from the drainage in case of CR-POPF occurrence. On the other hand, the *regret of the commission* referred to the regret of the surgeon who decided to place an IPD, resulting in useless action because the patients did not develop CR-POPF.

The regret of omission was measured through the following question: *“How would you rate the level of your regret, on a scale of 0 to 100 (0* = *no regret, 100* = *maximum regret) if you decided NOT to place the intraperitoneal prophylactic drain and the patient developed after DP a clinically relevant POPF requiring CT—percutaneous drainage?”*. Regret of the commission was elicited as follows: *“How would you rate the level of your regret, on a scale of 0 to 100 (0* = *no regret, 100* = *maximum regret) if you decided to place the intraperitoneal prophylactic drain and the patient had, after DP, normal postoperative course without clinically relevant POPF?”*.

In the regret model, Mt represents the POPF threshold at which regret of omission equals the regret of commission: Mt = (1/[1 + (regret of omission/regret of commission)]) × 100. In other words, Mt is the probability of clinically relevant POPF at which we are indifferent between 2 management strategies. If the expected CR-POPF rate is above the threshold, the regret of not placing IPD (omission) will be larger than the regret of placing them (commission). Hence, we should place IPD to minimize regret. The expected CR-POPF rate after DP was extracted from the proposed D-FRS for DP [12].

### Statistical analysis

Frequencies and percentages were used to describe categorical data. For continuous measures, mean, standard deviation (SD), median, and interquartile (IQR) ranges were used for continuous values. Age, gender, professional level, hospital type, the main activity of the surgical unit, implementation of minimally invasive PD (MIPD), type and number of drainage, the timing for drain removal, tailored strategy for the low and high-risk pancreatic remnant, perceived importance of closed system, drain mobilization, drain placement in preventing POPF grade B and C were tested in predicting regret of omission, commission and CR-POPF threshold. For these analyses, multilevel multivariate mixed-effects models were used. In these models, the geographic area of the participants was considered fixed because the study was not interested in regional differences. In other words, the total regression line represents the average Italian centers, independently from geographic origin. The effect of covariates was measured, and the coefficient and SE were reported. Post-estimation mean regrets and threshold were calculated for each category. A *p*-value <0.05 indicates a non-negligible effect on the regrets or threshold. Statistical analyses were performed with Stata (Stata Statistical Software: Release 15, StataCorp, LLC, College Station, TX).

## Results

### Participants

The survey started on July 08, 2023, and was closed on August 31, 2023. One hundred six surgeons completed the online survey. At the time of the survey, 143 surgeons were registered in AISP and AICEP, and the engagement rate was 74.1%.

In Table 1, the general information of respondents is reported.

**Table 1:** Characteristics of 106 participants

Characteristics of participants	*N* (%) or Median (IQR)
Sex	
Female	18 (17)
Male	88 (83)
Age, years	46 (36–57)
Professional Level	
Resident/Fellow	12 (11.3)
Attending	94 (88.7)
Geographic area	
North of Italy	71 (67)
Center of Italy	16 (15.1)
South of Italy	19 (17.9)
Hospital type	
Public, non-academic	25 (23.6)
Private, non-academic	7 (6.6)
Private, academic	19 (17.9)
Public, academic	55 (51.9)
Hospital volume of pancreatic resection, yearly	
<10	5 (4.7)
11–20	14 (13.2)
21–30	17 (16)
31–40	9 (8.5)
41–50	10 (9.4)
51–100	25 (23.6)
>100	26 (24.5)
Type of surgical unit	
Colorectal	6 (5.7)
Hepato-biliary	38 (35.9)
Pancreatic	32 (30.2)
General surgery, including all sub-specialties	30 (28.3)
MIDP	
No	11 (10.3%)
Yes	95 (89.7%)

*IQR* interquartile range, *MIPD* Minimally Invasive Pancreaticoduodenectomy

### Use and management of drainage

The use and management of drainage are reported in Table 2. Most surgeons (46.2%) declared using passive suction placing Easy Flow or Penrose drains. The closed system (with or without active suction) was the second most used (37.7%). Robinson drainage was used only by 15.1% of participants. Fifty-nine percent of respondents affirmed placing one drain. A change of strategy in high-risk pancreatic remnants was declared by 12.3% of respondents, increasing the number of drains. A change of strategy in low-risk pancreatic remnants was declared by 9.4% of respondents, reducing the number or omitting IPDs. Regarding the early removal, only 33% removed the drainage within the third POD if the drain fluid amylase criteria were satisfied. The median perceived importance of a closed system in preventing CR-POPF grade B was 3 (1–5, IQR); the perceived importance of passive suction in mitigating CR-POPF grade B was 4 (2–7, IQR); the perceived importance of drain in preventing CR-POPF grade C was 6 (3–8, IQR).

**Table 2:** Survey results about the use of drains after pancreaticoduodenectomy

Characteristics	*N* (%) or Median (IQR)
Type of drainage	
Easy flow/Penrose drainage	49 (46.2)
Robinson drainage	16 (15.1)
Jackson-Pratt or Blake drainage, with closed system and active suction	19 (17.9)
Jackson-Pratt or Blake drainage, with closed system and without active suction	21 (19.8)
Others	1 (0.9)
Number of drainages routinely used	
No	1 (0.9)
One	62 (58.5)
Two	43 (40.6)
*Change of strategy in high-risk pancreatic remnant*	
*No*	93 (87.7)
*Yes*	13 (12.3)
*Change of strategy in low-risk pancreatic remnant*	
*No*	96 (90.6)
*Yes*	10 (9.4)
*Change of strategy in MIDP*	
*No*	95 (89.6)
*MIDP not performed*	11 (10.4)
Timing of removal ^	
<=III POD	35 (33)
IV–V POD	49 (46.2)
VI–VII POD	15 (14.2)
>VII POD	7 (6.6)
*Perceived importance of closed system in preventing CR-POPF grade B*	3 (1–5)
*Perceived importance of passive suction in mitigating CR-POPF grade B*	4 (1–5)
*Perceived importance of drain in preventing a re-intervention*	6 (3–8)

*IQR* interquartile range, *FRS* Fistula Risk Score, *POD* Postoperative days, *CR-POPF* Clinically Relevant Postoperative Pancreatic Fistula; ^= in case of negative values of amylase, well general condition of patients and absence of suspicious fluid in the drainage

### Regret analysis

**Fig. 1:**
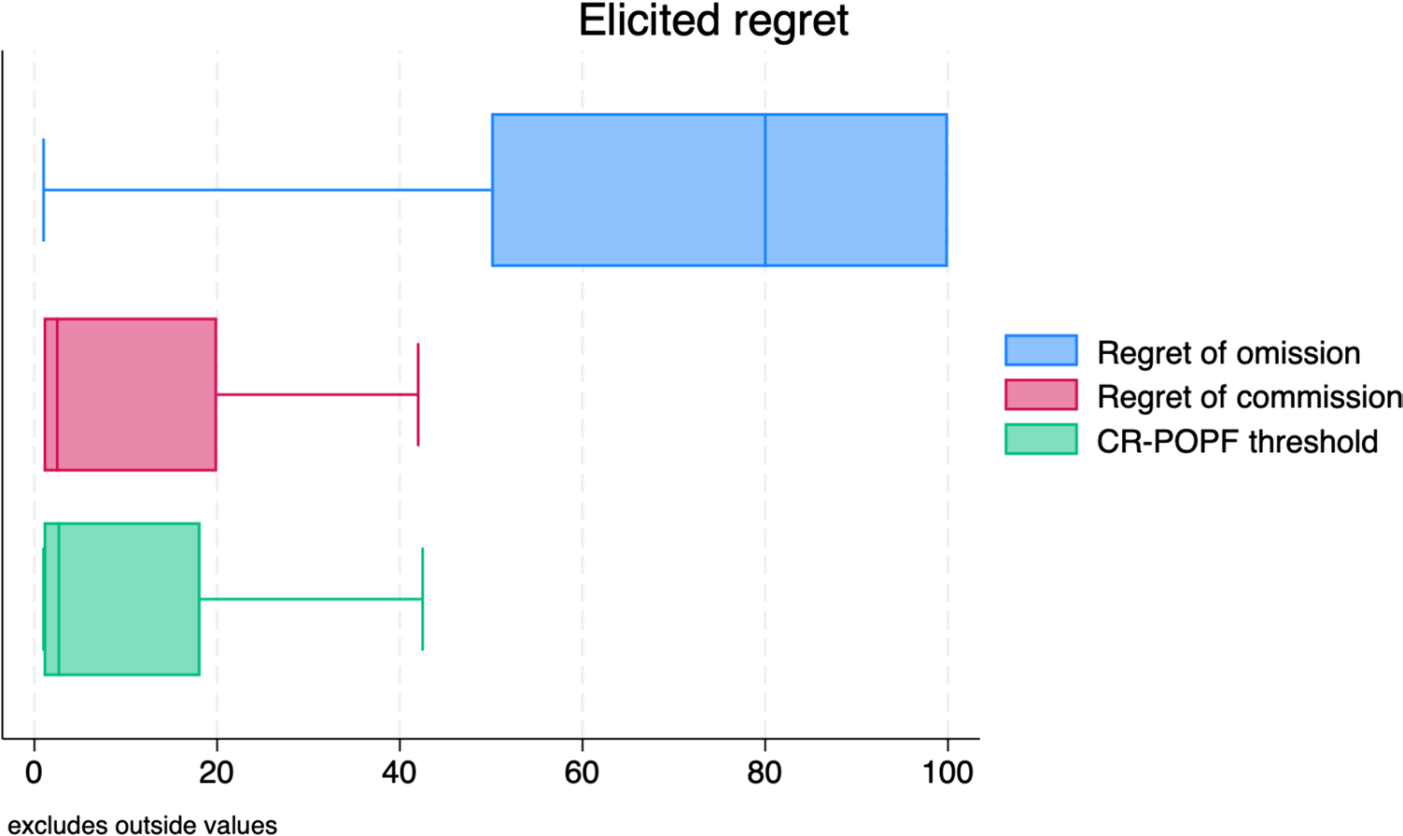
Box plots reporting regret of omission, commission, and CR-POPF threshold in the clinical vignette presented to the 106 respondents

Figure 1 shows regret of omission, commission, and CR-POPF risk threshold at which the omission is the least regrettable choice. The mean regret of omission was 69 (±33, SD), with a median of 80 (50–100, IQR). The mean regret of the commission was 15 (±22, SD) with a median of 2.5 (1–20, IQR). The mean CR-POPF risk probability threshold at which drainage omission was the less regrettable choice was 20(± 24) % with a median of 7% (1–35%, IQR). Multilevel effect multivariate regressions are reported in Supplementary Table 1, while the estimated mean of regrets and threshold is reported in Table 3.

**Table 3:** Postestimation values after multilevel mixed-effects multivariate regression

Covariates*	Regret of omission		Regret of commission		Risk threshold for CR-POPF^	
	Mean ± SD	*P*-value	Mean ± SD	*P*-value	Mean ± SD	*P*-value
Gender						
Male	71 ± 18	<0.001	14 ± 10	<0.001	14 ± 10	<0.001
Female	61 ± 16		20 ± 12		21 ± 12	
Volume						
Low-Medium^+^	77 ± 15	0.604	16 ± 8	0.194	17 ± 11	0.039
High	65 ± 19		15 ± 12		22 ± 15	
Prominent activity of surgical unit						
Pancreatic	58 ± 21	Ref.	19 ± 14	Ref.	19 ± 14	Ref.
Hepato-biliary	81 ± 12	0.047	16 ± 8	0.377	16 ± 8	0.458
Colo-rectal	60 ± 15	0.577	16 ± 11	0.599	16 ± 11	0.256
General surgery, including all sub-specialties	70 ± 14	0.165	11 ± 8	0.395	11 ± 7	0.214
MIDP						
No	59 ± 22	0.002	21 ± 11	0.297	33 ± 15	0.048
Yes	70 ± 18		15 ± 10		18 ± 13	
Type of drain						
Robinson, Jackson-Pratt, or Blake	73 ± 15	0.010	12 ± 8	0.341	16 ± 9	0.925
Easy Flow/Penrose	65 ± 21		18 ± 12		24 ± 17	
Type of system						
Open	67 ± 20	0.016	17 ± 11	<0.001	23 ± 15	<0.001
Close	74 ± 16		13 ± 9		16 ± 10	
Number of drains						
One	64 ± 18	<0.001	18 ± 11	0.020	24 ± 14	<0.001
Two	79 ± 14		11 ± 9		15 ± 12	
Custom to change strategy in low-risk pancreatic remnant						
No	72 ± 17	<0.001	14 ± 9	0.001	18 ± 12	<0.001
Yes	42 ± 13		33 ± 8			
Perceived importance of drain in preventing a re-intervention (VAS scale)	2 ± 2	0.173	−2 ± 1	<0.001	−2 ± 1	0.047

^*^=We only report covariates significantly affecting regret of omission, commission, or threshold; ° = Age, professional level, hospital type, active suction drain, custom to change strategy based on the high-risk, and perceived importance of drain utility in preventing CR-POPF or reintervention did not influence the results. ^ = The threshold indicates the CR-POPF risk rate at which the drain omission is the least regrettable choice; +  = inferior to 30 pancreatic resections per year; *MIDP* Minimally invasive distal pancreatectomy, *CR-POPF* Clinically Relevant Postoperative Pancreatic Fistula; § = the median values of visual analogic scale (VAS) are used to dichotomize the variables

**Fig. 2:**
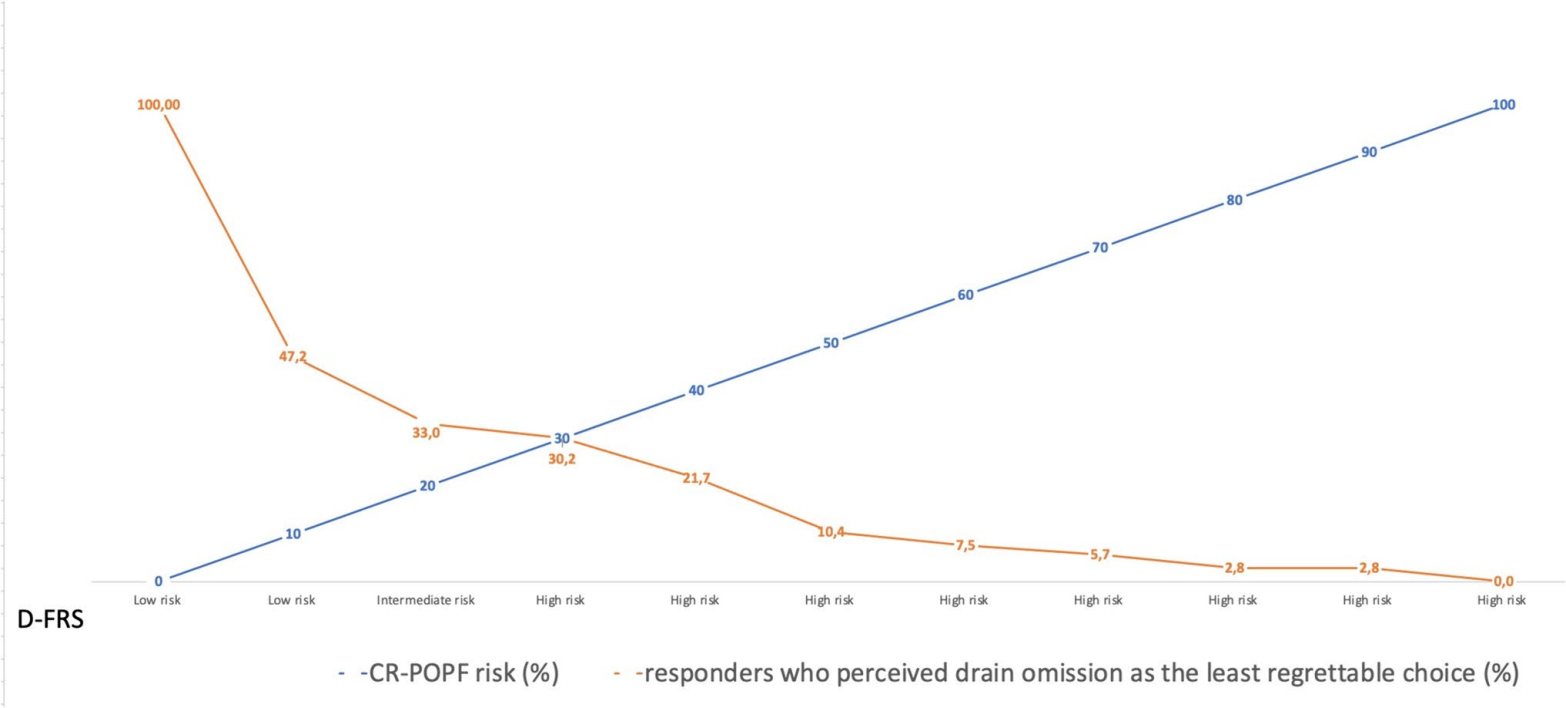
Percentage of responders who consider the IPD omission as the least regrettable choice based on the risk of CR-POPF. The x-axis represents the risk categories based on D-FRS. The blue line represents the linear risk of CR-POPF related to each category of D-FRS according to [11]; the orange line reports the percentage of responders who perceived the IPD omission as the least regrettable choice for the related risk of CR-POPF

In Fig. 2, we reported the percentage of respondents who perceived IPD omission as the least regrettable choice for each D-FRS risk category and the related probability of CR-POPF.

Supplementary Table 1 shows the multilevel mixed-effects regression. In Table 3, the estimated mean of regrets and threshold was reported. Age, professional level, hospital type, active suction preference, custom to change strategy based on the higher risk of CR-POPF, custom to remove the drain early, and perceived importance of drain closed system and passive suction in mitigating the CR-POPF did not affect regrets and threshold for CR-POPF.

Female surgeons had a significantly lower regret of omission (61 ± 16 vs. 71 ± 18; *P* < 0.001) and significantly higher regret of commission (20 ± 12 vs. 14 ± 10; *P* < 0.001). The threshold to accept the IPDs omission as the least regrettable choice was higher in female surgeons (21 ± 12 vs. 14 ± 10; *P* < 0.001). The volume of resections was related to the CR-POPF threshold: a high volume (>30 resections per year) was related to a high acceptability of IPD omission (22 ± 15 vs. 17 ± 11). Hepatobiliary surgeons showed a higher regret of omission than pancreatic surgeons (81 ± 12 vs. 58 ± 21; *P* = 0.047) without significantly affecting the CR-POPF threshold. The mean regret of omission was higher in MIDP surgeons than non-MIDP surgeons (70 ± 18 vs. 59 ± 22; *P* = 0.002). The final threshold for CR-POPF was 33 ± 15% vs. 18 ± 13% (*P* = 0.048), respectively. Surgeons who placed Easy Flow or Penrose drains (65 ± 21) had a significantly lower (*P* = 0.010) mean regret of omission than those who preferred Robinson, Jackson-Pratt, or Blake (73 ± 15) drains without significant effect on CR-POPF threshold. Participants who used a closed system had a higher regret of omission (74 ± 16 vs. 67 ± 20; *P* = 0.016) and a lower regret of commission (13 ± 9 vs. 17 ± 11; *P* < 0.001) and threshold for CR-POPF (16 ± 10% vs. 23 ± 15; *P* < 0.001).

Obviously, the surgeon who used more than one drain after DP experienced a higher regret of omission than those who placed only one or two drains (79 ± 14 vs. 64 ± 18; *P* < 0.001) and a lower regret of commission (11 ± 9 vs. 18 ± 11; *P* = 0.020) and CR-POPF threshold (15 ± 12 vs. 24 ± 14; *P* < 0.001). The survey participants who changed their strategy about IPD in low-risk pancreatic remnant have a lower mean regret of omission (42 ± 13 vs. 72 ± 17; *P* < 0.001), a higher regret of commission (33 ± 8 vs. 14 ± 9; *P* < 0.001), and CR POPF threshold (44 ± 13 vs. 18 ± 12; *P* < 0.001).

The higher the perceived importance of IPD’s role in preventing reoperation, the lower the regret of commission (*P* = < 0.001) and CR-POPF threshold (*P* = 0.047).

## Discussion

The present study showed that Italian pancreatic surgeons routinely use IPD after DP (99% of participants). Interestingly, only a minority of respondents declared that they would change their policy based on the characteristics of the pancreatic remnant. Moreover, only one-third of surgeons declared they would remove the drain early when CR-POPF could be excluded, even if some studies have demonstrated that early removal is possible in low-risk pancreatic remnants and when the level of amylase in the drain fluid is low [3]. Therefore, despite the availability, at the time of the survey, of at least one high-quality RCT [5] supporting the selective IPD omission, the current clinical practice seems to remain unchanged among the Italian pancreatic surgery community. The situation does not seem very different in other countries, and the selective IPD omission meets strong resistance worldwide. Indeed, the recent International guidelines about minimally invasive pancreatic surgery [13] did not provide clear recommendations on drainage omission after DP. This reluctance to IPD omission could be based on multiple reasons regarding the role of IPD after DP. The interviewed surgeons believed that IPD is important in preventing reintervention, scoring 6 out of 10 points.

On the contrary, other factors, such as passive suction or a closed system, seem to be marginal in the opinion of surgeons. For this reason, a certain degree of variability can be observed in the drain type preference through the Italian pancreatic surgical community. By the way, this heterogeneity was justified by literature evidence: at least two meta-analyses [14, 15] suggested that the type of drain or suction did not influence the rate and severity of CR-POPF.

In the present survey, we investigate the reasons for reluctance to adopt selective IPD omission using the “regret theory” approach. The regret methodology attributes a measurable value to emotional intelligence [16, 17]. A surgeon making a “non-repeatable” decision under uncertainty (e.g., omission of drainage) could experience regret in case of a negative result (e.g., CR-POPF), and this regret can be measured and used to optimize their choices. Indeed, the regret theory allows us to obtain the odd threshold for CR-POPF, at which the IPD omission was the least regrettable choice, as previously reported for PD [18]. In other words, we have tried to capture the pre-concepts or reasonable motifs that sustained the adoption or not of a tailored “drain-less” approach by asking the responders to elicit both regrets (commission and omission) in a real-life scenario. In this way, we have chosen to use a “low-risk” scenario because IPD omission can be considered safe only in this setting [5, 6].

The regret analysis showed some interesting results. First, the regret of omission was generally higher than that of the commission, producing a low CR-POPF threshold at which IPD omission was the least regrettable choice. In other words, the emotional intelligence of pancreatic surgeons also supported the IPD omission in low-risk scenarios. The median value of the CR-POPF risk threshold was 7%, while we previously reported a threshold value of 3% for PD in the same population of surgeons [18]. These data suggested that, at the same risk, selective IPD omission was perceived as a more reasonable choice in DP than PD. The reason for this perception is probably based on the theory of retrograde infection in which a biochemical leak was converted into a CR-POPF due to external contamination. While this theory seems reasonable for DP [19], it has been partially rebutted for PD [20]. The multivariate analysis confirmed this observation: the surgeons who used the closed system experienced lesser regret in placing a drain after DP because they probably believed that the closed system was a strategy similar to IPD omission in avoiding retrograde contamination. For this reason, the surgeons who used a closed system have a CR-POPF threshold lower than others in adopting IPD omission as the least regrettable choice.

Other reasonable factors influence the choice of IPD omission. First, female surgeons seem to have a better feeling about the selective IPD omission policy. This is not surprising because the effect of gender in decision-making, at least in the surgical area, is well-documented [21, 22]. Second, CR-POPF threshold regret was reduced in high-volume centers probably because the combination of expertise and resources, such as interventional radiology [23] and operative endoscopy [24], permits the management of the clinically relevant fluid collections without large regret even if IPD has been omitted. On the other hand, emotional intelligence suggested that, in the absence of resources able to treat undrained CR-POPF, the IPD omission should be avoided. Third, it is more difficult to accept a selective IPD omission when a surgeon routinely places two abdominal drains and when the risk of CR-POPF does not influence the policy. In this scenario, the threshold CR-POPF at which the IPD omission is considered the least regrettable omission is so low that this choice was rare. This attitude is partially justified. For many years, in the case of PD, a validated and worldwide accepted tool to predict the risk of CR-POPF has been available [8], while an FRS was only recently developed for DP and still requires full external validation [11, 12]. Moreover, if the surgeon perceived the IPD as an instrument to mitigate the risk of reoperation, the omission would be accepted only in very low-risk scenarios. However, this choice appears related to personal belief and is not supported by the evidence. At least two RCTs [5, 6] have demonstrated that the risk of surgical reintervention was not increased by selective IPD omission after DP. Finally, it should be noted that the survey underlines a well-known problem in the Italian healthcare system, in which most pancreatic centers are located in the North of the country [25].

The current study has limitations and strengths. The interviewed group was heterogeneous, including low and high-volume centers, academic and non-academic institutions, and dedicated and undedicated pancreatic surgeons. It is possible that not all Italian pancreatic surgeons are involved in this survey, even if most HPB surgeons are affiliated with AISP and AICEP. At the same time, this is a strength of the study because this survey represents a snapshot of the “real life” of the Italian pancreatic surgeon community. Moreover, each surgeon reported their habits based on personal and center experience.

These data conflict with recent evidence about the “drain-less” approach or early removal. Nonetheless, this survey may represent the starting point for a quality improvement evidence-based program. Another limitation is that we assumed for this study that a single decision-maker is involved in IPD omission or commission. At the same time, drain management is usually a shared surgical team decision, even if the survey was addressed to a single surgeon. In other words, the opinion of single surgeons could not always reflect the overall policy of the center. Finally, we did not plan a specific question for the day of amylase measurement. Some surgeons could not fully adopt the recommendation for ISGPF, such as the dosage of amylase from the third postoperative day. This could represent a source of heterogeneity for some questions, such as those related to early removal.

In conclusion, this survey showed that, despite available high-quality data that could justify IPD omission, especially in lower-risk scenarios, a certain reluctance to abandon the dogmas of “always drain” exists. This reluctance appears related to both rational and non-rational factors. A logical reason could be that a closed system can prevent retrograde infection, similar to the “drain-less” approach. On the other hand, an example of a non-rational reason is that reintervention could be avoided by placing IPD. Nonetheless, emotional intelligence could help the surgeons: i) to understand that an IPD omission is possible in low-risk scenarios without significant regret; ii) to address the educational program in eradicating “non-evidence-based” customs precluding “a priori” a risk assessment based selective drain policy.

## Supplementary Information

Below is the link to the electronic supplementary material.

Supplementary file1 (DOCX 24 KB)

## Data Availability

Data are available on request, to the corresponding author.
