# Correction: Italian survey about intraperitoneal drain use in distal pancreatectomy

**DOI:** 10.1007/s13304-024-02059-z

**Published:** 2024-12-12

**Authors:** Nicolò Pecorelli, Claudio Ricci, Alessandro Esposito, Giovanni Capretti, Stefano Partelli, Giovanni Butturini, Ugo Boggi, Alessandro Cucchetti, Alessandro Zerbi, Roberto Salvia, Massimo Falconi, Pan-Drain study group, Alberici Laura, Aleotti Francesca, Alfieri Sergio, Angrisani Marco, Anselmo Alessandro, Bannone Elisa, Barabino Matteo, Belfiori Giulio, Belli Andrea, Belli Giulio, Bonatti Chiara, Borgia Gianluca, Caccamo Lucio, Campra Donata, Caputo Damiano, Casadei Riccardo, Cescon Matteo, Citterio Davide, Colangelo Ettore, Colledan Michele, Coppola Roberto, Crippa Stefano, Dall’Olio Tommaso, De Carlis Luciano, De Giorgi Donato, De Luca Raffaele, Del Vecchio Antonella, Della Valle Raffaele, Di Benedetto Fabrizio, Di Dato Armando Di Domenico Stefano, Di Meo Giovanna, Di Sebastiano Pierluigi, Ettorre Giuseppe Maria, Fogliati Alessandro, Frena Antonio, Gavazzi Francesco, Giacomo Batignani, Gianotti Luca, Giuliante Felice, Grazi Gianluca, Grottola Tommaso, Gruttadauria Salvatore, Ingaldi Carlo, Isabella Frigerio, Izzo Francesco, La Barba Giuliano, Langella Serena, Lionetto Gabriella, Lombardi Raffaele, Maganuco Lorenzo, Maggino Laura, Malleo Giuseppe, Manzini Lorenzo, Marchegiani Giovanni, Marchetti Alessio, Marcucci Stefano, Massani Marco, Mastrangelo Laura, Mazzaferro Vincenzo, Mazzola Michele, Memeo Riccardo, Milanetto Anna Caterina, Mocchegiani Federico, Moraldi Luca, Moro Francesco, Napoli Niccolò, Nappo Gennnaro, Nardo Bruno, Pacilio Carlo Alberto, Paiella Salvatore, Papis Davide, Patriti Alberto, Patrono Damiano, Prosperi Enrico, Puglisi Silvana, Ramera Marco, Ravaioli Matteo, Rocca Aldo, Ruzzente Andrea, Sacco Luca, Scialantrone Grazisa, Serenari Matteo, Tamburrino Domenico, Tatani Bruna, Troisi Roberto, Veneroni Luigi, Vivarelli Marco, Zanello Matteo, Zanus Giacomo, Zingaretti Caterina Costanza, Zironda Andrea

**Affiliations:** 1 Pancreatic Surgery Unit, Pancreas Translational and Clinical Research Center, San Raffaele Scientific Institute, Milan, Italy; 2 Vita-Salute” San Raffaele University, Milan, Italy; 3 Department of Internal Medicine and Surgery (DIMEC), Alma Mater Studiorum, University of Bologna, Bologna, Italy; 4 General and Pancreatic Surgery Department, The Pancreas Institute-University of Verona Hospital Trust, Verona, Italy; 5 Pancreatic Surgery Unit, Humanitas Clinical and Research Center-IRCCS, Via Manzoni 56, Rozzano, Milan Italy; 6 Department of Biomedical Sciences, Humanitas University, Via Rita Levi Montalcini 4, Pieve Emanuele, Milan, Italy; 7 Surgical Department, HPB Unit Pederzoli Hospital, Peschiera Del Garda, Italy; 8 Division of General and Transplant Surgery, University of Pisa, Pisa, Italy; 9 Morgagni E Pierantoni Hospital, Forlì, Italy

**Correction: Updates in Surgery** 10.1007/s13304-024-01987-0

In this article the author’s name Giovanna Di Meo was incorrectly written as Giovanni Di Meo under the acknowledgement collaborators list.

The original article has been corrected.

