# Supplementary material for: Italian survey about intraperitoneal drain use in distal pancreatectomy
Source: Updates Surg. 2024 Oct 13;77(1):9–17. doi: 10.1007/s13304-024-01987-0 (PMC11876192; doi:10.1007/s13304-024-01987-0)
Supplement: Supplementary file 1 — Supplementary file1 (DOCX 24 KB) [file 13304_2024_1987_MOESM1_ESM.docx]

**Supplementary Table 1 – Multilevel mixed-effects regression evaluating the effect of the covariates on the Regret of omission, commission, and CR-POPF threshold.**

| **Covariates *** | **Regret of omission** | | **Regret of commission** | | **Threshold for CR-POPF ^** | |
| --- | --- | --- | --- | --- | --- | --- |
|  | **Coefficient ± SE** | **p-value** | **Coefficient ± SE** | **p-value** | **Coefficient ± SE** | **P-value** |
| **Age** (for each year) | 0.3 ± 0.3 | 0.157 | -0.1 ± -0.2 | <0.001 | -0.1 ± 0.2 | 0.003 |
| **Gender** (Male vs. Female) | -9.4 ± 2.6 | <0.001 | 13.3 ± 2.5 | 0.894 | 9.5 ± -3.2 | 0.430 |
| **Professional Level** (Resident vs. Trainee) | -0.7 ± 8.9 | 0.938 | 5.7 ± 7.8 | 0.465 | 0.9 ± 11.4 | 0.934 |
| **Hospital** **type** (Teaching vs. non-teaching) | -4.6 ± 3.2 | 0.157 | 2.9 ± 3.5 | 0.411 | 2.7 ± 2.8 | 0.344 |
| **Hospital type** (Public vs. Private) | 1.8 ± 7.1 | 0.800 | 2.5 ± 3.4 | 0.465 | 1.6 ± 3.2 | 0.606 |
| **Hospital volume** (Low-Medium vs. High) | 0.8 ± 1.5 | 0.604 | -3.9 ± 3.1 | 0.194 | -3.2 ± 1.6 | 0.039 |
| **Prominent activity of surgical unit**  Pancreatic  Colorectal  Hepato-biliary  General surgery, including all sub-specialties | 1.0 (referent)  15.6 ± 8.1  5.2 ± 12.5  4.9 ± 5.5 | 0.049  0.677  0.483 | 1.0(referent)  4.7 ± 5.3  -8.5 ± 16.1  -3 ± 3.5 | 0.377  0.599  0.395 | 1.0(referent)  -4 ± 5.4  - 4.9 ± 13.8  -2.7 ± 2.1 | 0.458  0.256  0.214 |
| **MIPD** (No vs. Yes) | 17.2 ± 5.5 | 0.002 | -7.9 ± 7.6 | 0.297 | - 21.1 ± 4.9 | <0.001 |
| **Type of drain** (Robinson, Jackson-Pratt, or Blake vs. Easy Flow/Penrose) | 4.4 ± 1.7 | 0.010 | 4.2 ± 4.4 | 0.341 | 0.4 ± 3.8 | 0.925 |
| **Type of system** (Open vs. Close) | 7.7 ± 3.2 | 0.016 | -5.2 ± 0.9 | <0.001 | -8.5 ± 1.4 | <0.001 |
| **Active suction** (No vs. Yes) | 3.1 ± 5.7 | 0.586 | 0.5 ± 5.1 | 0.924 | -0.5 ± 4.4 | 0.913 |
| **Number of drain** (One or two) | 19.9 ± 5 | <0.001 | -9.2 ± 4.1 | 0.020 | -14 ± 4.2 | 0.001 |
| **Change strategy in low-risk pancreatic remnant** (No vs. Yes) | -22.7 ± 4.7 | <0.001 | 25.1 ± 7.8 | 0.001 | 30.6 ± 5.1 | <0.001 |
| **Change strategy in high-risk pancreatic remnant** (No vs. Yes) | -0.8 ± 6.8 | 0.902 | -1.5 ± 2.9 | 0.599 | 0.7 ± 3.4 | 0.833 |
| **Timing for drain removal** (early vs. late) | 2.7 ± 4.9 | 0.584 | 0.1 ± 2.4 | 0.963 | 0.3 ± 3.2 | 0.938 |
| **Importance of closed system in preventing grade B CR-POPF** (for each point) | 1.1 ± 0.8 | 0.172 | 0.3 ± 1.5 | 0.856 | -0.7 ± 1.3 | 0.612 |
| **Importance of passive suction in mitigating grade B CR-POPF** (for each point) | -0.2 ± 1.1 | 0.844 | 0.1 ± 0.4 | 0.727 | 0.1 ± 0.5 | 0.759 |
| **Importance of drain in preventing re-intervention** (for each point) | 2.3 ± 1.7 | 0.173 | -1.9 ± 0.4 | <0.001 | -1.8 ± 1.1 | 0.047 |

**Legend**: *= geographic area was included in all models as fixed effect; ^= The CR-POPF risk rate at which the drain omission is the least regrettable choice, calculated with FRS; FRS= Fistula Risk Score; CR-POPF= Clinically Relevant Postoperative Pancreatic Fistula.
